# Reactive Oxygen Species and the Cardiovascular System

**DOI:** 10.1155/2013/862423

**Published:** 2013-04-22

**Authors:** Yannick J. H. J. Taverne, Ad J. J. C. Bogers, Dirk J. Duncker, Daphne Merkus

**Affiliations:** ^1^Department of Experimental Cardiology, Erasmus Medical Center Rotterdam, P.O. Box 2040, 3000 CA Rotterdam, The Netherlands; ^2^Department of Cardiothoracic Surgery, Erasmus Medical Center Rotterdam, P.O. Box 2040, 3000 CA Rotterdam, The Netherlands

## Abstract

Ever since the discovery of free radicals, many hypotheses on the deleterious actions of reactive oxygen species (ROS) have been proposed. However, increasing evidence advocates the necessity of ROS for cellular homeostasis. ROS are generated as inherent by-products of aerobic metabolism and are tightly controlled by antioxidants. Conversely, when produced in excess or when antioxidants are depleted, ROS can inflict damage to lipids, proteins, and DNA. Such a state of oxidative stress is associated with many pathological conditions and closely correlated to oxygen consumption. Although the deleterious effects of ROS can potentially be reduced by restoring the imbalance between production and clearance of ROS through administration of antioxidants (AOs), the dosage and type of AOs should be tailored to the location and nature of oxidative stress. This paper describes several pathways of ROS signaling in cellular homeostasis. Further, we review the function of ROS in cardiovascular pathology and the effects of AOs on cardiovascular outcomes with emphasis on the so-called oxidative paradox.

## 1. Introduction

In the mid 1950s, free radicals were first proposed to be involved in the pathophysiology of a number of diseases [1]. However, due to their short life span and the technical difficulty of detecting them, it took till the 1980s to recognize the importance of reactive oxygen species (ROS) as important players in biological systems [2]. Nowadays, it is widely accepted that ROS play a crucial physiological role, not only in various diseases, but also in cellular homeostasis [3].

ROS are chemically reactive molecules derived from molecular oxygen and formed as a natural by-product of aerobic metabolism. During energy conversion, ROS are produced as a by-product of oxidative phosphorylation, which is presumed to be the major source of superoxide (O_2_^•−^) production [4, 5]. ROS can also be produced through a variety of enzymes including xanthine oxidase and NAD(P)H oxidase [3]. 

Under normal circumstances, ROS concentrations are tightly controlled by antioxidants, keeping them in the picomolar range [3]. These low concentrations of ROS enable their role as second messengers in signal transduction for vascular homeostasis and cell signaling. When excessively produced, or when antioxidants are depleted, ROS can inflict damage onto lipids, proteins, and DNA. This intracellular reduction-oxidation imbalance, called oxidative stress, can subsequently contribute to the development and/or progression of cardiovascular diseases such as atherosclerosis, ischemia-reperfusion injury, chronic ischemic heart disease, cardiomyopathy, congestive heart failure, and even ensuing arrhythmias [2, 6–8].

Apparently, within cellular physiology there is a paradoxical role for ROS, which is temporally and spatially defined. In this paper we will discuss this dual role by summarizing the aspects of ROS generation and metabolization in the cardiovascular system, with focus on the role of ROS in cardiovascular cell signaling, in particular hydrogen peroxide (H_2_O_2_). In addition, we will discuss the role of ROS in ischemic heart disease.

## 2. Molecular Basis of ROS

ROS encompass free radicals, oxygen ions, and peroxides, both organic and inorganic, but all derived from molecular oxygen. They are formed as necessary intermediates in a variety of normal biochemical reactions [3]. Only when produced in excess or not appropriately controlled, they can inflict damage within the body. 

A division can be made into two groups: free radicals and other ROS. Free radicals have an extremely high chemical reactivity due to the unpaired free electron (i.e., superoxide anion O_2_^•−^ and hydroxyl radical OH^•^). Other ROS like H_2_O_2_ and peroxynitrite (ONOO^−^) are not considered free radicals as they lack the free unpaired electron and thus have oxidizing rather than reactive effects [3, 9].

### 2.1. Formation of O_2_^•−^

**Figure 1:**
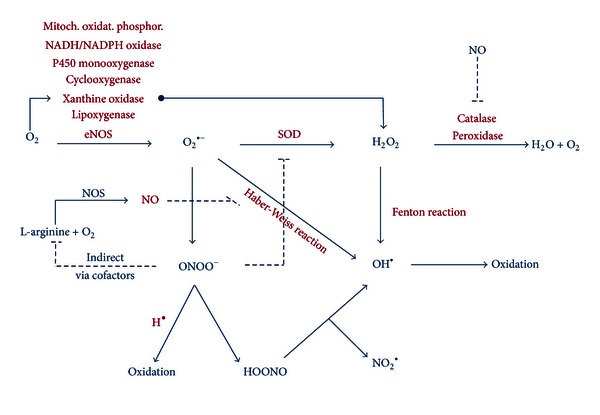
Summary of production and removal of various reactive oxygen species. Superoxide (O_2_^•−^) can dismutate in several ways, either spontaneously through a reaction with superoxide dismutase (SOD), through the Haber-Weiss reaction, or in reaction with nitric oxide (NO) and its radical (NO^•^). Through SOD, hydrogen peroxide (H_2_O_2_) is formed and further reduced by catalase and peroxidase to form water and oxygen. Also, H_2_O_2_ can be formed directly from xanthine oxidase. The hydroxyl radical (OH^•^) is formed through the Haber-Weiss reaction, through the Fenton reaction, and from peroxynitrous acid (HOONO). O_2_^•−^ can also scavenge NO to form peroxynitrite (ONOO^−^) leading to nitroso-redox imbalance. NADH/NADPH oxidase: nicotinamide adenine dinucleotide (phosphate); (e)NOS: (endothelial) derived nitric oxide synthetase; NO: nitric oxide, NO_2_: nitric dioxide; mitoch. Oxidat. phosphor, mitochondrial oxidative phosphorylation; dotted lines: inhibition.

Within living cells, O_2_^•−^ is produced through two distinct pathways, namely, enzymatically (Figure 1) and nonenzymatically. The latter occurs when a single electron is directly transferred to oxygen by reduced coenzymes or prosthetic groups (e.g., flavins or iron sulfur clusters).

For most tissues, the primary source of O_2_^•−^ is situated in the mitochondrial electron transport chain. It contains several redox centers that may leak approximately 1%-2% of the electrons to oxygen [5, 10]. Enzymatically, O_2_^•−^ is produced from a variety of substrates, through different enzymes, most importantly NAD(P)H (nicotinamide adenine dinucleotide phosphate) oxidases, xanthine oxidases, and endothelial nitric oxide synthase (eNOS), as will be discussed below [11].

NADPH/NADH oxidases, located on the cell membrane of polymorphonuclear cells, macrophages, and endothelial cells, play an important role in generation of O_2_^•−^ [3, 9]. Under normal circumstances, NAD(P)H oxidases catalyze the reaction of NAD(P)H, H^+^, and oxygen to form NAD(P)^+^and H_2_O_2_. These oxidases are mainly present in adventitial fibroblasts but in different vascular pathologies, such as atherosclerosis and hypertension; upregulation of NAD(P)H expression has been shown in endothelial and vascular smooth muscle cells [12].

The conversion of xanthine dehydrogenase to xanthine oxidase (XO) provides another enzymatic source of O_2_^•−^ and H_2_O_2_, which in turn constitutes a source of OH^•^. The relative amounts of O_2_^•−^ and H_2_O_2_ formed by XO depend on the conditions. At physiological oxygen concentrations between 10%, and 21%, H_2_O_2_ constitutes about 75% of ROS formed, whereas at lower oxygen concentrations, H_2_O_2_ formation from XO approaches 95% [13].

The oxidase itself converts hypoxanthine into xanthine and xanthine into uric acid. Normally this process accounts for a small part of the ROS production but under pathological conditions, it has been proposed to mediate deleterious processes in vivo. For example, after reperfusion, large quantities of XO are released into the circulation possibly reacting with plasma purine substrates and molecular oxygen to produce ROS [14]. Chemical reactions forming molecular oxygen through NAD(P)H and xanthine oxidases are summarized in (1)–(4), respectively.

(1) $$\begin{array}{c} \text{NAD(P)H}\;\;\text{oxidase}\;\;\text{reaction}:\\ \text{NAD(P)H}+2{\text{O}}_{2}\to {\text{NAD(P)}}^{+}+2{{\text{O}}_{2}}^{•-}+2{\text{H}}^{-} \end{array}$$

(2) $$\begin{array}{c} \text{Xanthine}\;\;\text{oxidase}\;\;\left(\text{XO}\right)\;\;\text{reaction}:\\ \text{Hypoxanthine}+{\text{H}}_{2}\text{O}+2{\text{O}}_{2}\to \text{Xanthine}+2{{\text{O}}_{2}}^{•-}+2{\text{H}}^{-} \end{array}$$

(3) $$\begin{array}{c} \text{Xanthine}+{\text{H}}_{2}\text{O}+2{\text{O}}_{2}\to \text{Uric}\;\;\text{acid}+2{{\text{O}}_{2}}^{•-}+2{\text{H}}^{-} \end{array}$$

(4) $$\begin{array}{c} \left(\text{Hypo}\right)\text{Xanthine}+{\text{H}}_{2}\text{O}+{\text{O}}_{2}\to \text{Urate}+{\text{H}}_{2}{\text{O}}_{2}. \end{array}$$

Within vascular pathology, a potential important source of O_2_^•−^ can be attributed to endothelial nitric oxide synthase [15]. There are three NOS isoforms, neuronal NOS (nNOS), endothelial NOS (eNOS), and inducible NOS (iNOS). In most cardiovascular tissues, nNOS and eNOS are constitutively present [3]. NOS enzymes normally catalyze the conversion of L-arginine to L-citrulline and produce nitric oxide (NO). eNOS is a heterodimer with both reductase and oxygenase domains on each monomer. In order to produce NO via eNOS, electrons must be transferred from the cofactor NAD(P)H to flavin adenine dinucleotide and flavin adenine mononucleotide to heme. The electron flow through eNOS to L-arginine, resulting in the production of NO, is dependent on the availability of its cofactors [16].

**Figure 2:**
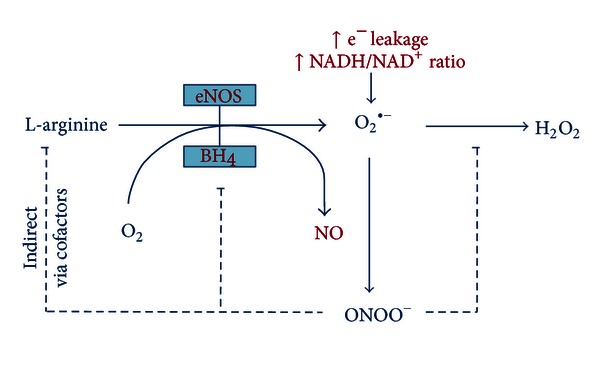
eNOS uncoupling. Excess superoxide (O_2_^•−^) production, for example, after myocardial infarction, results in scavenging of nitric oxide (NO) to form peroxinitrite. The latter inhibits coupling of not only endothelial derived nitric oxide synthetase (eNOS) and tetrahydrobiopterin (BH_4_), but also L-arginine and superoxide dismutase (SOD), which creates a downward spiral of enhanced O_2_^•−^ production. Finally, eNOS gets uncoupled and produces O_2_^•−^ rather than NO, sustaining the loop of nitroso-redox imbalance.

The balance between nitric oxide (NO) and O_2_^•−^ production is regulated by the availability of tetrahydrobiopterin (BH_4_). BH_4_ is involved in the catalytic process of L-arginine oxidation [17]. With impaired bioavailability of BH_4_, O_2_^•−^ is released rather than NO, a condition referred to as “eNOS uncoupling” [15] (Figure 2), where electrons that normally flow from the reductase domain to the heme group, now divert towards molecular oxygen rather than L-arginine [15, 18]. In vascular disease, a major part of this catalytic enzyme is uncoupled due to BH_4_ deficiency. The consequent increase in O_2_^•−^ rapidly reacts with NO to form peroxinitrite (ONOO^−^). ONOO^−^ oxidizes BH_4_ leading to “eNOS uncoupling,” and more production of O_2_^•−^, thereby creating a vicious circle of ROS induced ROS production [15]. As will be discussed later on, the resulting endothelial dysfunction disturbs normal vascular responses and is associated with the development of atherosclerosis [11]. Importantly, endothelial dysfunction has been shown to be a prognostic factor for progression of atherosclerotic disease as well as cardiovascular event rate [19].

### 2.2. Reduction of O_2_^•−^

Oxidation-reduction reactions are highly similar to acid-base reactions and concern the transfer of electrons. The key in these reactions is that electrons are exchanged between reaction partners and not shared as with covalent bindings. Oxidation-reduction reactions are matched set, meaning that for every oxidation reaction there is simultaneous reduction reaction and are therefore called “half-reactions.”

Oxidation refers to loss of electrons, while reduction denotes the gain of electrons. The change of electrons between partners can be predicted by using the oxidation number, which is the algebraic difference between the number of protons and electrons in a specific ion. The produced intermediates are able to oxidize (by donating electrons) several molecules. When oxygen is scarce, cells move to a more reduced state resulting in altered function of biomolecules. This redox signaling comprises oxireductive chemical reactions that alter proteins posttranslationally, thereby creating a coupling between redox state and cell function [6]. 

O_2_^•−^ can dismutate (be reduced) in several ways (Figure 1), either spontaneously through a reaction with superoxide dismutase (SOD), through the Haber-Weiss reaction, or in reaction with NO. O_2_^•−^ has a half-life of 10^−9^ to 10^−11^ s while in the presence of SOD, the half-life decreases to 10^−15^ s. The reaction catalyzed by SOD reduces two O_2_^•−^ radicals to form oxygen and H_2_O_2_ which in turn can be fully reduced to H_2_O and oxygen:

(5) $$\begin{array}{c} 2{{\text{O}}_{2}}^{•-}+2{\text{H}}^{+}\to {\text{H}}_{2}{\text{O}}_{2}+{\text{O}}_{2}. \end{array}$$

H_2_O_2_ is oxidized by peroxidase and catalase. It has a half-life of 10^−3^ s in the absence of catalase and 10^−8^ s in its presence. Alternatively, H_2_O_2_ can react with reduced transition metals—called the Fenton reaction—to form OH^•^ and OH^−^ or OOH^•^ and H^+^, when combined with Fe^2+^ or Fe^3+^, respectively [3]:

(6) $$\begin{array}{c} {\text{Fe}}^{2+}+{\text{H}}_{2}{\text{O}}_{2}\to {\text{Fe}}^{3+}+{\text{OH}}^{•}+{\text{OH}}^{-} \end{array}$$

(7) $$\begin{array}{c} {\text{Fe}}^{3+}+{\text{H}}_{2}{\text{O}}_{2}\to {\text{Fe}}^{2+}+{\text{OOH}}^{•}+{\text{H}}^{+}. \end{array}$$

The typical range for the iron dose is 1 part of Fe per 5–25 parts of H_2_O_2_. The optimal pH for the Fenton reaction is between 3 and 6. When the pH is too high, iron precipitates in Fe(OH)_3_ and will decompose H_2_O_2_ into oxygen. The Fenton reaction occurs predominantly at the endoplasmatic reticulum but not at mitochondria or other intracellular compartments [20]. Liu and coworkers showed that the Fenton reaction is involved in oxygen sensing, through regulation of genetic expression of hypoxia inducible factor-1 (HIF-1) [20]. An important role of HIF-1 is to establish the optimal balance between glycolytic and oxidative metabolism at any oxygen concentration to maximize ATP production without increasing ROS levels. Thus, HIF-1 induces metabolic reprogramming in cells that are oxygen deprived, thereby reducing mitochondrial respiration, minimizing O_2_^•−^ production [21], and contributing to a fast responding oxygen-sensing system.

The reaction of O_2_^•−^ with NO^•^, controlled by the rate of diffusion of both radicals, forms the very potent oxidant ONOO^−^. ONOO^−^ in turn is oxidized or reacts with a hydrogen radical (H^•^) to form the stable HOONO. The latter dismutates quickly into OH^•^ and free nitrogen species (NO_2_^•^). Thus concentrations of OH^•^ increase by means of H_2_O_2_ and HOONO dismutation (metal independent pathway). Alternatively, OH^•^ can be generated through the Haber-Weiss reaction, when superoxide radicals and H_2_O_2_ molecules spontaneously combine to form molecular oxygen and hydroxyl radicals:

(8) $$\begin{array}{c} {{\text{O}}_{2}}^{•-}+{\text{H}}_{2}{\text{O}}_{2}\to {\text{OH}}^{•}+{\text{OH}}^{-}+{\text{O}}_{2}. \end{array}$$

## 3. ROS and Cell Signaling

### 3.1. Signal Transduction Pathways of Cellular Responses to ROS

**Figure 3:**
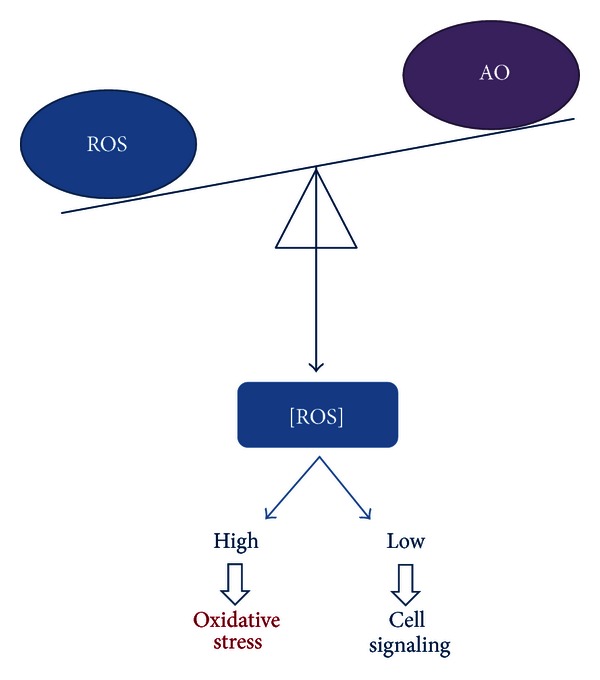
Redox balance. The production of reactive oxygen species (ROS) is tightly controlled by antioxidants (AOs) keeping the concentration of ROS ([ROS]) in the picomolar range. This low [ROS] is necessary for adequate cell physiology. When ROS is excessively produced or AOs are depleted, there is a high intracellular [ROS] leading to oxidative stress and resulting in cellular damage.

**Figure 4:**
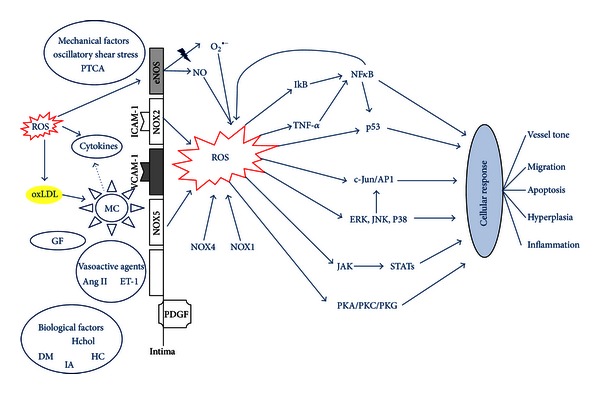
ROS production and vascular signaling. Several mechanical as well as circulating factors can increase ROS concentrations by acting on the tunica intima. The increased amounts of ROS activate specific second messenger systems which finally convey a cellular response. Hchol: hypercholesterolemia; DM: diabetes mellitus; IA: infectious agents; HC: homocysteine; MC: monocyte; GF: growth factors (PDGF, IGF-1, EGF, etc.); Cytokines (IL-1, TNF-*α*, etc.); oxLDL: oxidized low-density lipoprotein; eNOS: endothelial derived nitric oxide synthetase; PK: protein kinase A/C/G.

**Figure 5:**
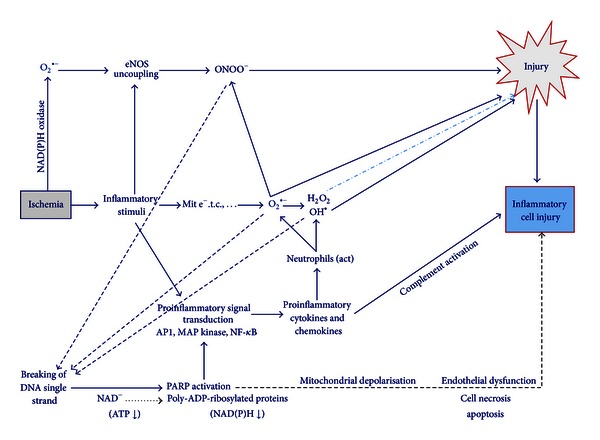
Different pathways leading to cell injury after ischemia. Ischemia due to atherosclerotic obstruction leads to an inflammatory process which provides the starting point of many other pathways of cellular injury via ROS production. Three main paths distinguished, being endothelial derived nitric oxide synthase (eNOS) uncoupling, mitochondrial electron transport, and proinflammatory signal molecules. Further, the produced ROS interact leading to DNA breaking and thus protein modification, with further cellular injury and dysfunction. Mit e^−^.t.c.: mitochondrial electron transport chain; ATP: adenosine triphosphate; NAD(P)H: nicotinamide adenine dinucleotide phosphate oxidase; PARP: poly-ADP-ribosylated proteins; O_2_^•−^: superoxide; H_2_O_2_: hydrogen peroxide.

Most cells have been shown to generate a small burst of ROS when stimulated by, for example, cytokines, angiotensin II (Ang II), endothelin-1 (ET-1), and platelet derived growth factor (PDGF) [22], leading to the hypothesis that ROS play an important role in cellular homeostasis and communication [3] (Figure 3). ROS signaling involves alterations in the intracellular redox state and oxidative modification of regulatory and contractile proteins (Figures 4–5).

The intracellular redox state within cellular homeostasis is primarily balanced by the glutathione/glutathione disulfide couple which functions as a major redox buffer, indicating/determining the redox state of the cell [23]. Glutathione (GSH) is abundantly present in the cytosol, nucleus, and mitochondria. It is synthesized in the cytosol and transported to the mitochondria and the nucleus [24]. GSH exhibits protection against ROS by (a) participating in amino acid transport through the plasma membrane, (b) scavenging OH^•^, H_2_O_2_, and lipid peroxidases via glutathione peroxidase (GPx) (catalytic reaction), (c) being a cofactor in numerous detoxifying enzymes (e.g., GPx), and (d) regeneration of the most important AOs back to their active form [25]. The latter function is linked with the redox balance of GSH with its oxidized form GSSG [26].

Glutathione exists in reduced (GSH) and oxidized (GSSG) states. When reduced, the thiol group of cysteine can donate a reducing equivalent to other unstable molecules such as ROS. By doing so, glutathione itself becomes reactive and quickly reacts with another reactive glutathione to form glutathione disulfide (GSSG). Once oxidized, glutathione can be reduced back by glutathione reductase, thereby using NAD(P)H as an electron donor. Under normal physiological conditions, more than 90% of the glutathione in the cell is in the reduced form (GSH) and less than 10% exists in the disulfide form. An increase in GSSG/GSH ratio, for example due to inactivation of glutathione reductase by ONOO^−^, is considered to be indicative of oxidative stress [27].

ROS can posttranslationally modify proteins. Redox signaling typically involves amino acid oxidation, hydroxylation, or nitration. Targets usually are redox sensitive cysteine residues within the proteins, which have a low ionization pKa of 4-5 compared to a pKa of 8.5 of nonreactive cysteines in other proteins [28, 29]. The modifications in these redox sensitive proteins alter their conformation, stability, activity and/or ability to interact with other proteins, resulting in modulation of cellular function. Redox sensitive proteins include proteins involved in calcium handling as well as contractile proteins, proteins involved in various signaling pathways and proteins involved in transcriptional activities. 

Redox modulation of calcium handling proteins directly affects cardiac contraction by altering intracellular calcium. Examples of redox sensitive calcium handling proteins are calcium calmodulin kinase II (CaMKII), the ryanodine receptor on the sarcoplasmic reticulum, sarcoplasmic reticulum ATPase (SERCA), and phospholamban (for review see [29, 30]). Moreover, the contractile proteins can also be oxidatively modified by oxidation or nitrosylation [30]. Typically, oxidation of contractile proteins is assumed to depress cardiac function, although recently some modifications have been identified that actually increase contractility. The current understanding on how oxidative stress modulates cardiac function is limited mostly because many studies have focused on isolated myofilament proteins whereas oxidative modifications of different contractile proteins occur simultaneously in vivo and act in concert. Hence, the contributions of the individual oxidative modification are difficult to establish [30].

The second group of proteins affected by direct redox modification are protein kinases and phosphatases. Since tyrosine phosphorylation is an early signaling event in many signal transduction pathways, alterations in activity through redox modification of protein kinases upstream in the signaling cascade result in indirect modulation of protein kinases more downstream in the cascade. Tyrosine phosphorylation in vascular smooth muscle cells is important in the control of vascular tone. Thus, tyrosine phosphatase inhibitors generally constrict smooth muscle, whereas tyrosine kinase inhibitors cause relaxation [28]. Oxidative modification results in inhibition of phosphotyrosine phosphatases (PTP 1A, PTP1B, and PTEN), while the protein kinase Src is activated by oxidation. Src has many targets in the cell. Interestingly, Src activates receptor tyrosine kinases such as the EGF-receptor in vascular smooth muscle cells. This activation occurs independent of EGF, and the activated receptor then acts as a signaling platform for the stimulation of phospholipase enzymes, production of lipid mediators, and activation of downstream kinases such as PI3K, Akt, ERK, and PKC [28].

PKC is directly activated by oxidation of the cysteine residues in its regulatory site, which occurs at low concentrations of oxidants. Conversly, PKC is inhibited by oxidation of cysteine residues in its catalytic domain, which occurs at higher concentrations of oxidants. Alterations in PKC activity affect many signaling cascades in the cell, including modulation of calcium sensitivity of the myofilaments and receptor tyrosine kinase signaling [28, 30]. The cAMP-dependent protein kinase A (PKA) and the cGMP-dependent protein kinase G (PKG) are also susceptible to redox modification. Both PKA and PKG are involved in regulation of vascular tone as well as cardiomyocyte contraction. When PKA oxidation occurs in its regulatory domain, it promotes dissociation of the catalytic and regulatory subunits resulting in cAMP independent PKA activation [29, 30]. However, similar to PKC, oxidation of cysteine residues in the catalytic subunit inhibits PKA activity [29]. Oxidation of PKG in its dimerization domain results in activation of the enzyme independently of the NO-cGMP pathway [29]. Oxidative modification of PKA, PKC, and PKG results in altered phosphorylation of the myofilaments, thereby modulating cardiac as well as vascular function.

The small monomeric G-proteins ras, rac-1, and RhoA are also activated by ROS. Activation of RhoA results in its translocation to the plasma membrane and activation of Rho-kinase. Rho-kinase appears to be a key player in cardiovascular function and cardiovascular pathology. Thus, in vascular smooth muscle cells, Rho-kinase regulates calcium sensitivity of the myofilaments via inhibition of myosin light chain phosphatase [31]. Moreover, activation of Rho-kinase contributes to smooth muscle proliferation, hypertrophy and motility [32]. Rho-kinase activation is responsible for upregulation of NAD(P)H oxidases, thereby contributing to a vicious circle of ROS, leading to Rho-kinase activation resulting in more ROS production. In endothelial cells, Rho-kinase negatively regulates NO-production both by destabilizing eNOS mRNA and through impairment of eNOS activity [31, 32], thereby also contributing to augmentation of oxidative stress. In cardiomyocytes the role of Rho-kinase is less well understood although it is thought to function in a similar way to its role in vascular smooth muscle. In addition, Rho-kinase is thought to be involved in cardiomyocyte hypertrophy and apoptosis [31]. Yet, the precise role of Rho-kinase, as well as its modulation by redox regulation in cardiac myocytes, remains to be determined.

Another group of kinases that are not directly redox sensitive but very important in cardiovascular cell signaling are mitogen-activated protein kinases (MAPKs) (Figure 4). MAPKs are indirectly activated by ROS via the ROS sensitive kinases Src, PKC, ras, and the MAPK kinase kinase ASK-1 [33]. MAPKs are divided into three subgroups: extracellular signal regulated kinases (ERKs): ERK1 and ERK2; c-Jun N-terminal kinases (JNKs): JNK1, JNK2, and JNK3; and p38 kinases: p38 *α*, *β*, *γ*, and *δ* [3, 34, 35]. In addition to being indirectly activated by ROS, MAPKs are activated by environmental stresses and inflammatory cytokines, which are also known to induce oxidative stress.

The third important group of redox sensitive proteins in the cardiovascular system are the proteins involved in transcriptional activity, not only including transcription factors but also histone deacetylases (HDAC). ROS can both inhibit and stimulate cellular NF-*κ*B signaling [36], while certain NF-*κ*B regulated genes play a major role in regulating the amount of ROS in the cell. Also, recently ROS have been shown to directly connect the important redox sensitive transcription factors NF-*κ*B and HIF-1, implicating a novel signaling pathway in cardiovascular pathology (Figure 5) [37].

Histone acetylation by histone acetylases promotes gene expression, while histone deacetylation by HDACs inhibits gene expression. Oxidation of HDAC4 and HDAC5 that are expressed in cardiac myocytes results in export of these HDACs from the nucleus, thereby inhibiting their activity. As these HDACs normally inhibit the transcription of prohypertrophic genese their oxidation may be involved in induction of hypertrophy [29].

The long-term consequences of ROS for cardiovascular (dys)function depend on the balance between signals promoting proliferation or growth inhibition and/or cell death. ROS can alter this balance leading to either excessive angiogenesis or loss of endothelial cells [2]. The dual role of ROS in “fine-tuning” the balance between apoptosis and excessive cell growth is illustrated by observations that, during ischemia-reperfusion injury, ROS trigger apoptosis, while ROS generated during ischemic preconditioning prevent apoptosis [38–41]. ROS generated during ischemic preconditioning are capable of upregulating expression of the Bcl-2 gene [42], which regulates the intrinsic pathway of apoptosis [43, 44]. This gene is also activated via the nuclear transcription factor NF-*κ*B and its activation has been shown to reduce apoptosis [42, 45].

Conversely, endothelial apoptosis initiated by tumor necrosis factor-*α* (TNF-*α*) and mediated by activation of JNK can be attenuated by ROS scavenging [46]. TNF-*α* has been implicated in inflammatory responses of the heart and vasculature. Thus, TNF-*α* is one of the inflammatory cytokines that are produced in the ischemic region and surrounding myocardium following myocardial infarction. Also, vessels from subjects with diabetes are characterized by an increased TNF-*α* production, increased ROS production, and endothelial dysfunction [47]. Similarly, endothelial dysfunction induced by advanced glycation end products (AGEs) is mediated through elevated TNF-*α* expression and induction of ROS production with NF-*κ*B functioning as the link between TNF-*α* and AGEs/RAGE signaling [48, 49]. TNF-*α* and NF-*κ*B are interrelated in that translocation of NF-*κ*B into the nucleus has been proposed to be pro-inflammatory and, either directly or indirectly, leads to a significant increase in TNF-*α* production while TNF-*α* activates NF-*κ*B, which then regulates genes involved in inflammation, oxidative stress, and endothelial dysfunction [49, 50]. Interestingly, ROS produced in response to TNF-*α* can further activate NF-*κ*B which again activates TNF-*α* creating a vicious circle [51]. Hence, ROS play a key role in the induction of vascular dysfunction in response to TNF-*α* [47].

### 3.2. The Role of H_2_O_2_ in Signal Transduction

H_2_O_2_ is an interesting molecule within the ROS family. It is a waste product of mitochondrial electron transfer and, hence to be created, no additional energy is required. The chemical properties of H_2_O_2_, such as a short half-life, rapid metabolization by catalase, and rapid reaction with thiols, are ideal properties for H_2_O_2_ to act as a signaling molecule.

H_2_O_2_ mediates diverse physiological responses including cell differentiation, proliferation, and migration, and has been proposed to be involved in metabolic vasodilation [3, 70]. In cells stimulated with growth factors and cytokines (PDGF, EGF, insulin, TNF-*α*, and interleukins), the NAD(P)H oxidase gp91 Phox and homologues form the major source of H_2_O_2_ [34, 70–72]. However, the coupling between receptor activation to the NAD(P)H oxidase complex (Nox) is still poorly understood [73]. In order to mediate different responses, H_2_O_2_ modifies the activity of key signaling proteins. It catalyzes redox reactions, oxidizing primarily cysteine residues of proteins thereby altering their function. For example, the activity of tyrosine phosphatases is H_2_O_2_ dependent. The chemical configuration of these phosphatases contains a cysteine and arginine site resulting in a low PK_a_ and existing as a thiolate anion. The latter is more susceptible to H_2_O_2_ oxidation which abolishes its activity and is reversed by cellular thiols [74]. Not only tyrosine phosphatase but also tyrosine kinase (Src) is oxidized by H_2_O_2_ [75]. An overall regulation must exist, that is, both temporal and spatial, ensuring process activation (only when and where needed) and termination after exerting its effects.

In order to induce protein alterations, H_2_O_2_ must increase rapidly above a certain threshold, but each cell contains natural AO enzymes. Therefore H_2_O_2_ has to be protected from destruction. H_2_O_2_ is inactivated by peroxiredoxin, which is part of a family of antioxidant enzymes whose thioredoxin peroxidase activity plays an important role in protecting against oxidative stress [76]. Interestingly, H_2_O_2_ causes hyperoxidation, and thereby inactivation, of peroxiredoxin, prolonging H_2_O_2_ bioavailability. The inactive peroxiredoxin can be reactivated by the adenosine triphosphate-dependent enzyme sulfiredoxin [77].

How H_2_O_2_ is actually delivered to the cytosol remains incompletely understood. H_2_O_2_ must cross the lipid bilayer towards the target molecules in the cytosol. Although it is generally assumed that H_2_O_2_ crosses the membrane freely, recent research indicates some membranes to be poorly permeable to H_2_O_2_ [78]. A shift in membrane lipid composition or a transport through aquaporins was presented as an alternative pathway to transfer H_2_O_2_ molecules to the cytosol [79]. Alternatively, H_2_O_2_ may also pass through gap junctions to exert its effect [80–82].

There is clearly a duality present in the role for H_2_O_2_ in cell proliferation. On the one hand, low concentrations of H_2_O_2_ play an important role in regulating cell growth, although the question remains if this effect is exerted merely through second messengers (JAK/STAT) or if H_2_O_2_ also has a direct effect on growth. On the other hand, high concentrations of H_2_O_2_ are responsible for cell apoptosis while moderate doses cause the cell to arrest in the G1 phase [83]. Recently, H_2_O_2_-induced apoptosis was shown to be mediated through a PKC-dependent pathway, antagonized by Akt and heme oxygenase-1 [84]. Also, recent data support the hypothesis that H_2_O_2_ can function as a transmitter of the apoptotic signal from the region of programmed cell death to neighboring healthy cells [85]. Apoptotic cells produce H_2_O_2_, thereby possibly contributing to the pathogenesis of, for example myocardial infarction and ageing. More specifically, mitochondrial produced ROS are needed for the generation of the apoptotic signal since specifically designed mitochondrial antioxidants (such as SkQs) inhibit this pathway [86].

H_2_O_2_ may also be involved in the response to vascular injury. Vascular smooth muscle cell (VSMC) death has been shown to occur after mechanical trauma like stenting. In response to vascular injury, potent chemotactic factors, such as bFGF, PDGF, TGF-*β*, and Ang II, are released [2]. These chemotactic factors in turn regulate VSM proliferation and migration. Two independent research groups have shown that this release of chemotactic factors is H_2_O_2_ dependent and thus reduced when administrating a scavenger like catalase [87, 88]. The hypothesis for ROS involvement in VSMC proliferation is further supported by a study showing that catecholamine induced VSCM proliferation can be blocked by N-acetylcysteine, tiron (superoxide scavenger), and diphenylene iodonium [89]. To allow VSMC migration and vascular remodeling, degradation of the extracellular matrix is required, which is partially accomplished by matrix metalloproteinases (MMPs). Both activity and expression of MMP-2 and MMP-9, the two MMPs thought to be most important in vascular remodeling, have been shown to be regulated by the nitroso-redox balance. Thus, H_2_O_2_ and OONO^−^ increase MMP-2 activity while reduction of MMP-2 and MMP-9 can be obtained by overexpressing eNOS [90, 91]. Not only the activity of the MMP family is modulated by ROS but also their expression, thereby providing a dual mechanism for ROS to regulate vascular remodeling. 

### 3.3. ROS and Pathophysiology (Figures 4 and 5)

The mitochondrial respiratory chain is one of the most prominent cellular sources of ROS. Hence, ROS production is related to oxygen consumption, making cells with high oxygen consumption more prone to oxidative stress. Oxygen consumption is particularly high in cardiac myocytes that are therefore equipped with a high number of mitochondria and a high level of respiratory chain components per milligram of mitochondrial protein. To prevent oxidative damage, these cells contain enzymatic scavengers of ROS such as SOD, glutathione peroxidase, catalase, and coenzyme Q10. Possibly together with nonenzymatic AO, they neutralize the deleterious effects of ROS. Mitochondria also possess the ability to repair themselves after oxidative damage using enzyme systems like phospholipid hydroperoxide glutathione peroxidase (PHGP). PHGP is a selenium containing enzyme directly reducing peroxidized acyl groups in phospholipids [92]. However, under pathological conditions, these protective mechanisms may fall short and make the cardiac myocytes vulnerable to oxidative damage.

With increased production of ROS, damage can be inflicted directly via oxidative modification of redox sensitive proteins [3, 10]. Also, inflammation, which in turn stimulates the release of O_2_^•−^, leads to cell injury, either directly or by depleting the natural AOs. An overview of the systems leading to cell damage via NO^•^, ONOO^−^, O_2_^•−^, H_2_O_2_/OH^•^, complement activation, and PARP activation is presented in Figures 3, 4, and 5. 

Some examples of how oxidative stress is involved in cardiac and vascular pathologies are described in the following sections.

### 3.4. Hypoxia, Ischemia, and Reperfusion

Medical strategies treating acute myocardial infarction require restoration of blood flow to the ischemic region. Unfortunately, this reperfusion is associated with a burst of ROS, which may continue for hours [93], and recruitment of inflammatory cells [94, 95]. These high levels of ROS cause structural damage of the heart, capillary leak, and influence cardiomyocyte metabolism thereby impairing both systolic and diastolic function [96]. Furthermore, not only ischemic damage, but also reperfusion, can produce dysfunction of the cardiac conduction system leading to arrhythmias [7, 97]. Besides increased ROS production, hypoxia, ischemia, and reperfusion have also been found to reduce levels of SOD, GSH, glutathione peroxidase, and ascorbate [98]. Hence, reduced scavenging further contributes to development of oxidative stress.

Reperfusion also inflicts damage on the vascular endothelium, with alterations in blood cells and microembolization, as well as vascular compression due to myocyte swelling, leading to changes in endothelial structure and alignment. The duration of ischemia is an important determinant of the extent of reperfusion damage. Ischemia-reperfusion damage can be reduced by ischemic preconditioning [99]. The mechanisms underlying this protection by ischemic preconditioning are incompletely understood [100]. Yet, a role for ROS as triggers for and mediators of this protective phenomenon has been consistently demonstrated. ROS can trigger preconditioning by causing activation of the mitochondrial K_ATP_ channel, which then induces generation of ROS and NO that are both required for preconditioning induced protection [101–104]. Importantly, ischemic preconditioning can be mimicked by administration of free radical donors S-morpholinosydnonimine [105] and even ONOO^−^ [106] while preconditioning can be blocked by free radical scavengers.

Ischemic preconditioning is therefore a clear example of the so-called oxidative paradox: AOs not only reduce deleterious ROS accumulation but also molecules necessary for cardioprotection.

### 3.5. Atherosclerosis

The majority of cardiovascular disease is a direct consequence of atherosclerosis. The transfer of oxidized low-density lipoprotein (ox-LDL) from the vessel lumen into the tunica media is regarded as the initiator for atherosclerosis at sites with endothelial damage [107]. Mechanical factors like fluid shear stress patterns play an important role in maintenance of endothelial function and initiation of endothelial dysfunction [3]. Thus, laminar shear stress induces expression of AO genes and production of NO^•^, preventing apoptosis and monocyte adhesion [108]. Branched arteries exposed to oscillatory shear stress are prone to atherosclerosis. This type of flow leads to continuous NAPDH-dependent production of O_2_^•−^ [109, 110]. Increased O_2_^•−^ can subsequently uncouple eNOS, thus creating an extra source of O_2_^•−^ production and leading to a vicious circle of ROS-induced ROS production. Upregulation of adhesion molecules (VCAM-1, E-selectin, P-selectin, and ICAM-1) [111] at locations with disrupted flow patterns is also ROS dependent and is further enhanced by cytokines like interleukins, TNF-*α*, Ang II, and vascular endothelial growth factor (VEGF) [3, 109–111]. Upregulation of adhesion molecules facilitates adherence and transmigration of leucocytes (Figures 4 and 5). Once converted to macrophages, they are capable of producing much higher amounts of ROS. ROS convert ox-LDL into highly oxidized LDL which itself is engulfed by these macrophages, forming foam cells and initiating the formation of the fatty streak.

Overall, the amount and pattern of blood flow are very important for endothelial function where aberrant flow patterns predispose to ROS production and atherosclerosis.

### 3.6. Clinical Evidence for Therapeutic Use of Antioxidants?

The deleterious effects of ROS can be reduced by restoring the imbalance between production and clearance of ROS [112]. Gey and colleagues found low rates of cardiovascular disease (CVD) in people consuming AO rich diets [113]. This second line of defense includes nonenzymatic antioxidant substances from dietary intake such as ascorbic acid (vitamin C), *α*-tocopherol (vitamin E), GSH, flavonoids, carotenoids, and others. In vitro studies indicated oxidation inhibition of low-density lipoproteins (LDL) by a number of these nonenzymatic AOs. Exogenous therapeutic administration of antioxidants has therefore been proposed as therapy for oxidative stress and cardiovascular disease. Despite some promising effects of such AO administration (Table 1), particularly in animal studies, caution should be warranted as these results could usually not be reproduced in clinical trials. Thus, conflicting results on the use of dietary supplementation of AOs, especially vitamin C, vitamin E, *β*-carotene, and selenium, have been presented (Table 2). AO supplementation is potentially deleterious for normal “redox homeostasis.” Not only is the redox balance very delicate, but also ROS play important roles in cell signaling and are therefore essential for survival of the organism [3, 9, 114]. The most relevant AOs used in dietary supplementation are flavonoids and vitamins C and E. The beneficial cardiovascular effects of these substances may not be limited to their AO effect, as they also include anti-inflammatory, platelet inhibitory, and antithrombotic effects.

One of the most studied AO supplements in prevention of cardiovascular disease is vitamin E. Many studies suggest a protective role for vitamin E, which has led to a massive marketing of vitamin E supplements. Conversely, large randomized studies (Table 2) could not substantiate this role for vitamin E. Indeed, meta-analysis failed to find a cardio protective effect nor did it find a reduction of clinical events in high risk patients or in patients with established disease [115–117]. Interestingly, a recent analysis even reports an increased mortality after using supplements of *β*-carotene and vitamin E [117].

When comparing Table 1 with Table 2, clinical trials fail to show a protective effect of AOs in humans. Not only are the positive outcome studies largely outnumbered by trials with no effects, they are also, in most cases, lacking the statistical power to be conclusive. The reason for failure of these AOs in clinical practice is most likely multifactorial. 

First of all, it is very difficult to detect subjects with a comparable imbalance between ROS production and AO defenses. Second, biochemical aspects inherent to each substance used, posology, and intake ratio must be taken into account. The lack of knowledge of the optimal dosage and route of administration for the various AOs is a serious limitation. For example, the bioavailability of vitamin C is determined by the availability of its transporter in the small intestine, and an increase of oral administration of vitamin C can actually decrease bioavailability [118]. This example illustrates that pharmacodynamical and pharmacokinetical properties of each AO should be known to optimize their application. To be effective, it is imperative that AOs reach the specific compartments of the cell where ROS are generated. For many vascular cells, this requires uptake into the cytoplasm (or vesicles) [2]. Importantly, AOs can become oxidants in some cellular compartments.

Third, lack of clinical improvement may be attributed to the selection of the population. Most patients enrolled in the clinical trials have already established CVD and in these cases AO therapy may be too late to be effective since, in animal studies, most protocols describe administration of AOs prior to the initiation of the disease. Finally, and perhaps most important, the choice of AOs should be based on the identity and location of ROS responsible for pathology. So instead of experimenting with cocktails of AOs in human trials, basic research should focus on targeting the specific pathways of different ROS responsible for the given pathology [112, 119].

The lack of cardiovascular benefit of AOs that are presently available has initiated research on new and more effective compounds. Clinical studies show that while these novel compounds do not reduce endpoints related to atherosclerosis, they improve endothelial function by increasing local NO bioavailability and therefore endothelium-dependent vasodilatation. Promising new agents are NO-donor phenols [120] and AGI-1067 that inhibits pro-inflammatory gene expression [121]. Other potentially promising AOs act through targeting NAPDH oxidases, Nox (VAS2870), and Nox2 peptide (gp91-dstat), preventing eNOS uncoupling or inhibiting xanthine oxidase (allopurinol) [2, 72, 119]. Technological developments also allow discovery of new functions for existing AOs. For example, oxidative damage of DNA was shown to be repaired in cells by naturally occurring phenols independent of known DNA repair enzymes thereby entailing possible new approaches [122].

Moreover, targeting AO therapy at specific sites of ROS production may be more effective in treatment of cardiovascular disease than global AO therapy [123]. Mitochondrial ROS scavenging is effective in treating hypertensive rats [124] and led to the realization that mitochondria play a central role in the pathogenesis of cardiovascular disease. New pharmacological approaches enable targeting of therapeutic substances at the mitochondria [125]. In particular, AOs conjugated with triphenylphosphonium cation such as mitoquinone (MitoQ), mitovitamin E, and mitophenyltertbutyline achieve much higher concentrations in the mitochondrial membrane, than those in the cytosol, due to the negative membrane potential of the inner mitochondrial membrane [126]. A new type of compounds, named SkQs, consisting of an antioxidant moiety (plastoquinone) and a penetrating cation, has been synthetized. This group of AOs specifically prevented oxidation of mitochondrial cardiolipin, arrested H_2_O_2_-induced apoptosis, and blocked necrosis initiated by ROS [86, 127]. Furthermore, SkQs also appear very promising in inhibiting the development of age-related diseases [86, 128]. However, so far, no studies have been performed that target mitochondria in cardiovascular disease in humans.

## 4. Conclusion

ROS play a dual role in cardiovascular (patho)physiology. ROS signaling plays an important part in endothelial function, vascular tone, and cardiac function. Conversely, when excessively produced, ROS can disrupt cellular signaling and inflict cellular damage. It thus appears that concentration and location of ROS are the main determinants of their function. 

Due to their very short half-life and technical difficulties of measuring ROS in vivo, little is known about the “safe margins” of ROS concentrations in cell signaling. Therefore, it is difficult to estimate which part of ROS production contributes to cellular homeostasis and normal physiological functioning and when ROS production becomes excessive and thereby detrimental. 

Although the deleterious effects of ROS can potentially be reduced by restoring the imbalance between production and clearance of ROS through administration of AOs, the dosage and type of AOs should be tailored to the location and nature of oxidative stress. Continuous administration of AOs in vivo can be unfavorable for normal cell signaling which, at least partially, explains the lack of clinical evidence on the beneficial actions of AO administration. 

New research should focus on matching AO therapy to oxidant stress present in the cardiovascular system. In vitro studies are extremely important to obtain knowledge on the mechanisms of oxidative damage as well as potential repair mechanisms, and when extrapolated to the in vivo setting with caution, they are likely to contribute to improve the therapeutic strategies for cardiovascular disease.

## Figures and Tables

**Table 1:** Major studies with possible beneficial effects of AOs on cardiovascular outcomes in humans.

Author/study	Journal	Design/FU	Population*	Agents (dosage/day)	Results
Stephens et al./CHAOS [52]	The Lancet	DB, PC 1.4 y (3 d–3 y)	*N* = 2002; M and F; mean 61.8 y; ischemic heart disease patients; secondary prevention	E (800 mg or 400 IU)	↓↓ I nonfatal MI, trend ↑ CV death
Duffy et al. [53]	The Lancet	DB, PC 30 d	*N* = 45; M and F; mean 48.5 y; HT patients	C (500 mg)	↓ BP in otherwise healthy HT
Boaz et al./SPACE [54]	The Lancet	DB, PC 2 y	*N* = 196; M and F; 40–75 y; haemodialysis patients	E (800 IU)	↓↓ combined endpoint of AMI = CV death + stroke
Neri et al. [55]	Clinical Therapeutics	DB, PC 15 d	*N* = 46; M and F; mean 40 y; DM/glucose intolerance patients	NAC (600 g) + C (250 mg) + E (300 mg)	↓ OS and inflammation
Accini et al. [56]	Nutrition, Metabolism and Cardiovascular Diseases	DB, PC 4 m	*N* = 57; M and F; 23–65 y; dyslipidemic patients	E (4 mg); PUFAn-3 (6602 mg EPA + 440 DHA); niacin (18 mg); *γ*OZ (40.2 mg)	↓ OS and inflammation markers

The CHAOS study is the largest study to report a strong decrease in nonfatal MI but, conversely, a slight increase in cardiovascular death. Other studies were performed in smaller groups. Overall, no overwhelming positive effects could be found in the studies. Population*: *N*: number of patients; M: male; F: female; y: age in years. DB: double blind; PC: placebo controlled; d: days; m: months; E: vitamin E; C: vitamin C; NAC: N-acetylcysteine; PUFAn-3: polyunsaturated fatty acids n-3; EPA: eicosapentaenoic; DHA: docosahexaenoic; *γ*OZ: *γ*-oryzanol; I: incidence; BP: blood pressure; CV: cardiovascular; MI: myocardial infarction; HT: hypertension; AMI: acute myocardial infarction; OS: oxidative stress.

**Table 2:** Major studies with no beneficial effects of AOs on cardiovascular outcomes.

Author/study	Journal	Design/FU	Population∗	Agents (dosage/day)	Results
Hennekens et al./physician health [57]	The New England Journal of Medicine	DB, PC, 2 × 2 12 y ( *β*C)	*N* = 22.071; all M, 40–84 y; former or current smokers	*β*C (50 mg) on alt days	No effect on CV death, AMI, or all-cause mortality
Rapola et al. [58]	The Lancet	DB, PC 5.3 y	*N* = 1862; all M; 50–69 y; smokers with previous MI	E (50 mg) + *β*C (20 mg)	No ↓ of MCE, ↑ risk FCHD
Virtamo et al. [59]	Archives of Internal Medicine	DB, PC 6.1 y	*N* = 27.271; all M; 50–69 y; smokers, no MI history; primary prevention	E (50 mg) + *β*C (20 mg)	E: ±↓ I fatal CHD, no ↓ I nonfatal CHD; *β*C no effect
Italiano/GISSI Prevenzione Investigators [60]	The Lancet	OL, PC, 2 × 2 3.5 y	*N* = 11.234, M and F; stratified for all age groups; AMI within 3 months; secondary prevention	E (600 mg) + fish oil (10 mg)	E: no effect AMI + death + stroke, fish oil: ↓ AMI + death + stroke
Yusuf/HOPE [61]	The New England Journal of Medicine	DB, PC, 2 × 2 4.5 y	*N* = 9451; M and F; ≥55 y; high risk CD patients; primary and secondary prevention	E (800 mg or 400 IU) Ramipril	E: no effect AMI + CV death + stroke; Ramipril: ↓ AMI + CV death + stroke
De Gaetano/PPP [62]	The Lancet	OL, PC, 2 × 2 3.6 y	*N* = 4495; M and F; mean 64.4 y; high risk CD patients; primary prevention	E (300 mg) Aspirin(100 mg)	E: no effect Aspirin: ↓ AMI + CV death + stroke
Collins et al./HPSCG [63]	The Lancet	DB, PC 5 y	*N* = 20.563; M and F; 40–80 y; CD, other OAD, DM patients	C (250 mg) + E (600 mg) + *β*C (20 mg)	No ↓ 5 y mortality
Törnwall et al. [64]	European Heart Journal	DB, PC 5–8 y	*N* = 29.133; all M; 50–69 y; smokers with risk on MCE or MI history	E (50 mg) or *β*C (20 mg) or both	*β*C: ↑ risk nonfatal MI; E: no effect
Armitage et al./HPS [65]	BMC Medicine	DB, PC, 2 × 2 5.5 y	*N* = 20.536; M and F; 40–80 y; high risk CD patients; primary and secondary prevention	Simvastatin (40 mg) C (250 mg) + E (600 mg) + *β*C (20 mg)	AO: no effect
Cook et al./WACS [66]	Archives of Internal Medicine	DB,PC, 2 × 2 9.4 y	*N* = 8171; all F; ≥1 CVE in history, secondary prevention	C (500 mg) + E(600 IU) on alt days + *β*C (50 mg) on alt days	No effect AMI + CV death + stroke + morbidity
Lee et al. [67]	Journal of The American Medical Association	DB, PC, 2 × 2 10.1 y	*N* = 39.876; all F; >45 y, healthy.	E (600 IU) Asiprin (100 mg)	No benefit for major CV events. No effect on total mortality
Lonn/HOPE II [68]	Journal of The American Medical Association	DB, PC 7.0 y	*N* = 3994, >55 y with vascular disease or DM; extension of HOPE I trial.	E (400 IU)	No prevention of major CV events. No prevention of cancer. Risk of HF may be ↑.
Kataja-Tuomola et al. [69]	Annals Medicine	DB, PC, 2 × 2 6.1 y	*N* = 29.133, all M smokers, some with DM.	E (50 mg/d) *β*C (20 mg/d)	No protective effect on macrovascular outcomes or total mortality.

Large multicenter studies all presented the same result that oral AOs had no beneficial effect on cardiovascular outcomes. Some studies even showed an increased risk of coronary heart disease. Population*: *N*: number of patients; M: male; F: female; y: age in years; 2 × 2: 2 × 2 factorial design comparing placebo, agent A, agent B, and combination of agent A and B; DB: double blind; PC: placebo controlled; E: vitamin E; C: vitamin C; *β*C: beta-carotene; FCHD: fatal coronary heart disease; MCE: myocardial event; CHD: coronary heart disease; AMI: acute myocardial infarction; CV: cardiovascular; OS: oxidative stress; IU: international units; HF: heart failure.
